# ENGAGING AND INVOLVING PEOPLE LIVING WITH DEMENTIA IN RESEARCH: WHAT TO (NOT) EXPECT

**DOI:** 10.1093/geroni/igac059.1463

**Published:** 2022-12-20

**Authors:** Martina Roes

**Affiliations:** German Center for Neurodegenerative Diseases (DZNE), Witten, Nordrhein-Westfalen, Germany

## Abstract

Collaborating across disciplines, professions, and countries as well as engaging a variety of stakeholders has been a goal for researchers for a long time. Interestingly, when people living with dementia and their significant others are actively involved and become a co-researcher or a member of a patient advisory board, the perspective of what collaboration and/or engagement and involvement means, becomes blurry. The intersection between the individual reality of people living with dementia and researchers can be understood as (a) a door that opens up and leads to new (multiple) perspectives and understandings or (b) a disaster because misconceptions of dementia hinder mutual trust building. This presentation will provide examples on how to build trust and how sustain a relationship over the course of time (and progression of the disease). Since living with dementia is highly stigmatized this presentation will also reflect on the impact of misconceptions of dementia on engagement.

